# Enhanced preferential cytotoxicity through surface modification: synthesis, characterization and comparative in vitro evaluation of TritonX-100 modified and unmodified zinc oxide nanoparticles in human breast cancer cell (MDA-MB-231)

**DOI:** 10.1186/s13065-016-0162-3

**Published:** 2016-04-01

**Authors:** Biplab KC, Siddhi Nath Paudel, Sagar Rayamajhi, Deepak Karna, Sandeep Adhikari, Bhupal G. Shrestha, Gunjan Bisht

**Affiliations:** Department of Biotechnology, School of Science, Kathmandu University, Dhulikhel, Nepal; Department of Chemical Science and Engineering, School of Engineering, Kathmandu University, Dhulikhel, Nepal

**Keywords:** ZnO nanoparticles, Surface modification, Triton X, Cytotoxicity

## Abstract

**Background:**

Nanoparticles (NPs) are receiving increasing interest in biomedical research owing to their comparable size with biomolecules, novel properties and easy surface engineering for targeted therapy, drug delivery and selective treatment making them a better substituent against traditional therapeutic agents. ZnO NPs, despite other applications, also show selective anticancer property which makes it good option over other metal oxide NPs. ZnO NPs were synthesized by chemical precipitation technique, and then surface modified using Triton X-100. Comparative study of cytotoxicity of these modified and unmodified NPs on breast cancer cell line (MDA-MB-231) and normal cell line (NIH 3T3) were carried out.

**Results:**

ZnO NPsof average size 18.67 ± 2.2 nm and Triton-X modified ZnO NPs of size 13.45 ± 1.42 nm were synthesized and successful characterization of synthesized NPs was done by Fourier transform infrared spectroscopy (FT-IR), X-Ray diffraction (XRD), transmission electron microscopy (TEM) analysis. Surface modification of NPs was proved by FT-IR analysis whereas structure and size by XRD analysis. Morphological analysis was done by TEM. Cell viability assay showed concentration dependent cytotoxicity of ZnO NPs in breast cancer cell line (MDA-MB-231) whereas no positive correlation was found between cytotoxicity and increasing concentration of stress in normal cell line (NIH 3T3) within given concentration range. Half maximum effective concentration (EC50) value for ZnO NPs was found to be 38.44 µg/ml and that of modified ZnO NPs to be 55.24 µg/ml for MDA-MB-231. Crystal violet (CV) staining image showed reduction in number of viable cells in NPs treated cell lines further supporting this result. DNA fragmentation assay showed fragmented bands indicating that the mechanism of cytotoxicity is through apoptosis.

**Conclusions:**

Although use of surfactant decreases particle size, toxicity of modified ZnO NPs were still less than unmodified NPs on MDA-MB-231 contributed by biocompatible surface coating. Both samples show significantly less toxicity towards NIH 3T3 in concentration independent manner. But use of Triton-X, a biocompatible polymer, enhances this preferentiality effect. Since therapeutic significance should be analyzed through its comparative effect on both normal and cancer cells, possible application of biocompatible polymer modified nanoparticles as therapeutic agent holds better promise.

**Graphical abstract:**
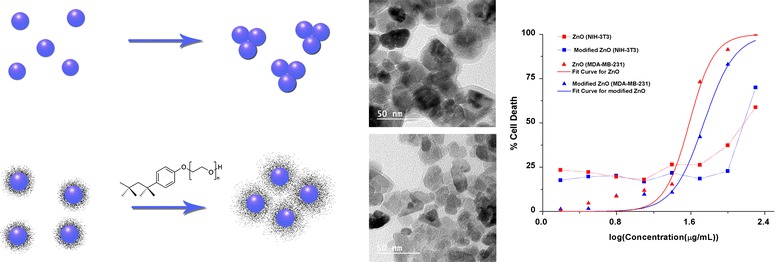
Surface coating, characterization and comparative in vitro cytotoxicity study on MDA-MB 231 and NIH 3T3 of ZnO NPs showing enhanced preferentiality by biocompatible surface modification.

## Background

Anticancer therapies relying on chemical, biological and natural products are not showing promising results because of their similar toxic effect on both normal proliferating cells and cancerous cell [1]. Hence, search for efficient and selective treatment for cancer has been a keen area of interest for most researchers which lead to selective targeting, delivery vehicles and selective agents engineering. The nanoscale magnitude and high surface area to volume ratio of NPs allow them to rework their characteristic properties permitting them to interact with biomolecules in a distinct way [2]. This property has increased the possibility of surface engineering according to need in cancer therapy, cell imaging, bio-sensing and drug delivery. Use of surfactant will reduce particle size but also alter the surface property of NPs [3]. Non-ionic polymers like TritonX, Tween 20, PEG, etc. have been widely used to make biocompatible surfaces for enhancing activity in biological environment in both delivery and therapeutic agents [4]. With extensive studies of anticancer activity of various metal oxide NPs, ZnO NPs, despite its other explicit applications on cosmetic, nanofabric and electronics [5], also shows selective killing of cancerous cell [6]. Although effect of surfactant on size and morphology of ZnO NPs is well characterized [7] and researches are also focused on explaining possible mechanism on preferential cytotoxicity [8, 9], study on significance of surfactant altered modification of these NPs on change in cytotoxicity of cancer and normal cells lines from unaltered one is still lacking. TritonX-100 being non-ionic biocompatible surfactants consisting both hydrophilic polyethylene oxide chain and hydrophobic aromatic group, has excellent detergent property, wetting ability and biodegradability [10]. Hence, in congruence with above facts and findings, this research aims to study the effect of surface altered ZnO NPs by TritonX-100 on preferential cytotoxicity in cancer cell invitro by comparing with innate preferential toxicity shown by unaltered ZnO NPs.

## Results and discussion

### Mechanism of synthesis

Zinc acetate (Zn(CH_3_COO)_2_) is soluble in methanol giving colorless solution. When methanolic solution of NaOH, a strong base is added dropwise to colorless ZnAc solution, white precipitate of Zn(OH)_2_ is formed. Upon adding excess concentrated NaOH, Zn(OH)_2_ dissolve to give Zincate (Zn(OH)_4_^2−^) ion at stoichiometric ratio. Under vigorous stirring considerable extent of Zincate dissociates into Zn^++^ and OH^−^ ions which upon reaching critical concentration forms, ZnO precipitates. Because of higher solubility of Zn(OH)_2_ as Zincate than ZnO in such condition, the reaction is favoured towards formation of ZnO [11].

$$ {\text{Zn}}\left( {{\text{CH}}_{ 3} {\text{COO}}} \right)_{ 2} + {\text{ NaOH}} \mathop{\longrightarrow}\limits^{{\text{Methanol}}/{\text{Vigorous}}\,{\text{Stirring}}}{\text{Zn}}( {\text{OH}})_{ 2} + {\text{ 2CH}}_{ 3} {\text{COONa}} $$

$$ {\text{Zn}}\left( {\text{OH}} \right)_{ 2} + {\text{excess NaOH}} \mathop{\longrightarrow}\limits^{{{\text{Methanol}}/{\text{Vigorous}}\;{\text{Stirring}}}} 2 {\text{Na}}^{ + } + {\text{ Zn}}\left( {\text{OH}} \right)_{ 4}^{ 2- } $$

$$ {\text{Zn}}\left( {\text{OH}} \right)_{ 4}^{ 2- } \mathop{\longrightarrow}\limits^{{{\text{Vigorous}}\,{\text{Stirring}}}}{\text{Zn}}^{ 2+ } + {\text{ 2OH}}^{ - } {\text{ZnO }} + {\text{ H}}_{ 2} {\text{O}} $$

**Fig. 1:**
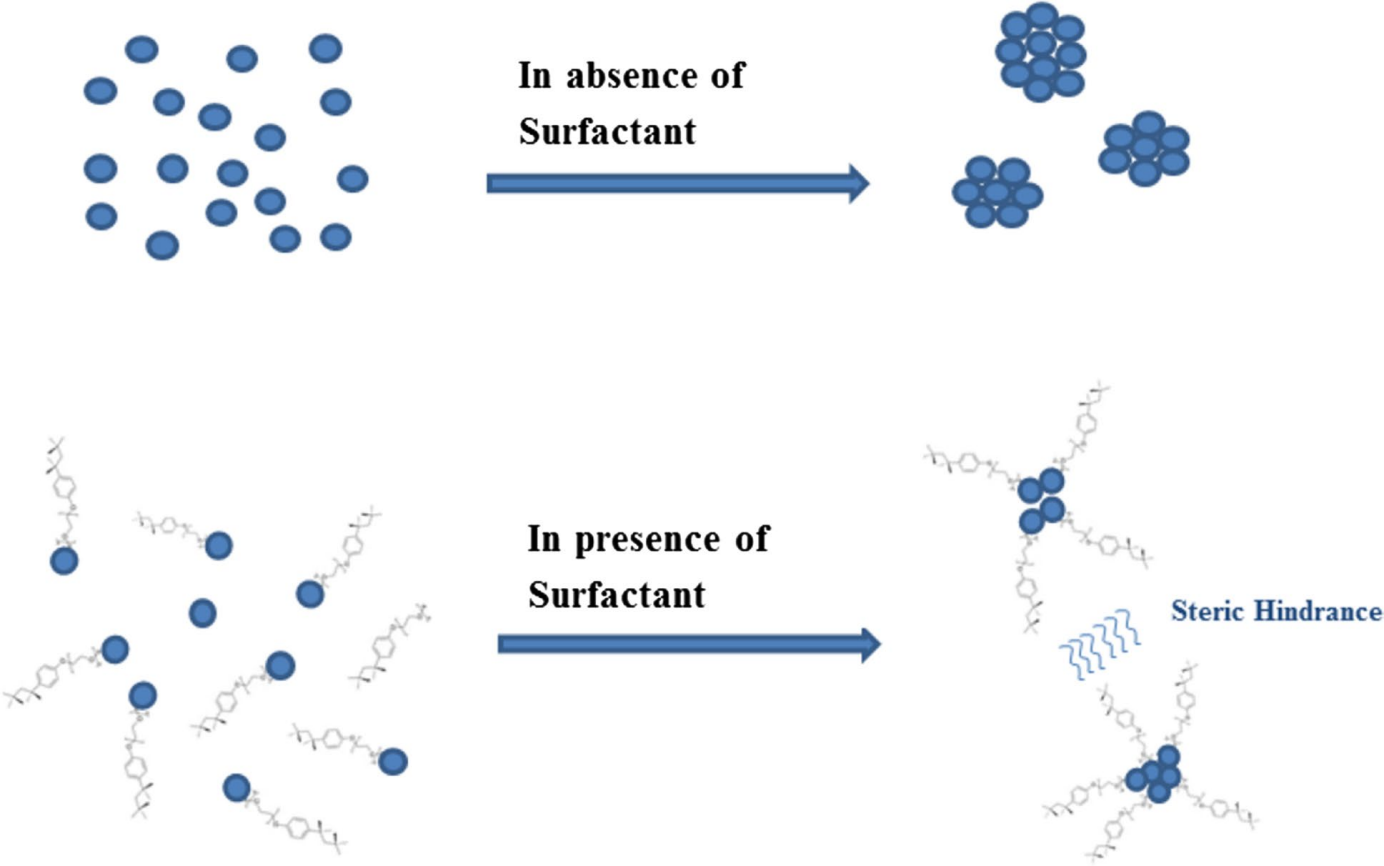
A hypothetical model of surfactant interaction with ZnO nucleus during particle growth

Subequent washing by distilled water removes excess base and sodium salts from the precipitate. Use of surfactant generally reduce particle size by interacting with formed nucleus and hindering other nucleus to come nearby during particle growth phase as shown in Fig. 1 as a hypothetical model in which bulkier hydrophobic group of TritonX-100 will restrict the free collision of ZnO nucleus during particle growth [3].

### Structural analysis

**Fig. 2:**
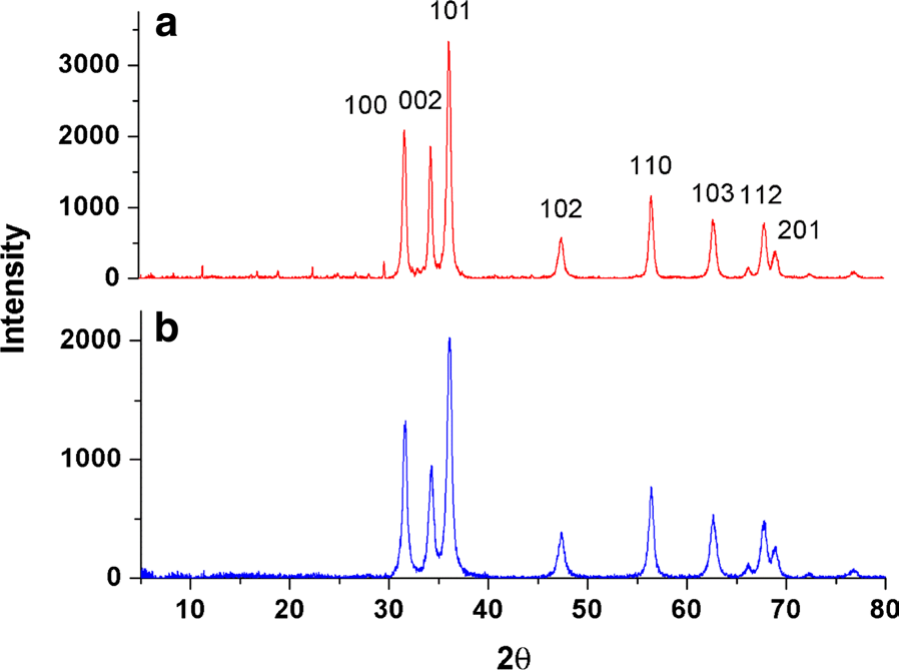
XRD peaks of ZnO (**a**) and modified ZnO (**b**) showing peaks at 2θ values from 5° to 80° with corresponding values showing miller indices (hkl values)

As it can be observed from Fig. 2, ZnO NPs (modified ZnO NPs) show sharp diffraction peaks corresponding to hkl values of 100, 002, 101 and 110 at 2θ values of 31.765 (31.693), 34.391 (34.308), 36.195 (36.112) and 56.606 (56.401) respectively pointing out to crystalline nature. Average particle size was obtained as 18.67 ± 2.2 nm for ZnO NPs and 13.45 ± 1.42 nm for modified ZnO NPs using Scherrer’s equation. Relative intensities for modified ZnO NPs are less than that of unmodified ZnO NPs which could be due to coating of non-crystalline TritonX. Corresponding miller indices obtained from Powder X software indicate crystalline planes of polygonal Wurtzite structure of ZnO(A) [12]. The decrease in particle size in modified ZnO could be due to possible coating during synthesis process where exposed bulky groups provide steric hindrances for nucleus agglomeration. Since particle size also depends on calcination period and time [13, 14], use of same parameters for both samples verify that the formation of reduced grain size is contributed by use of surfactant.

**Fig. 3:**
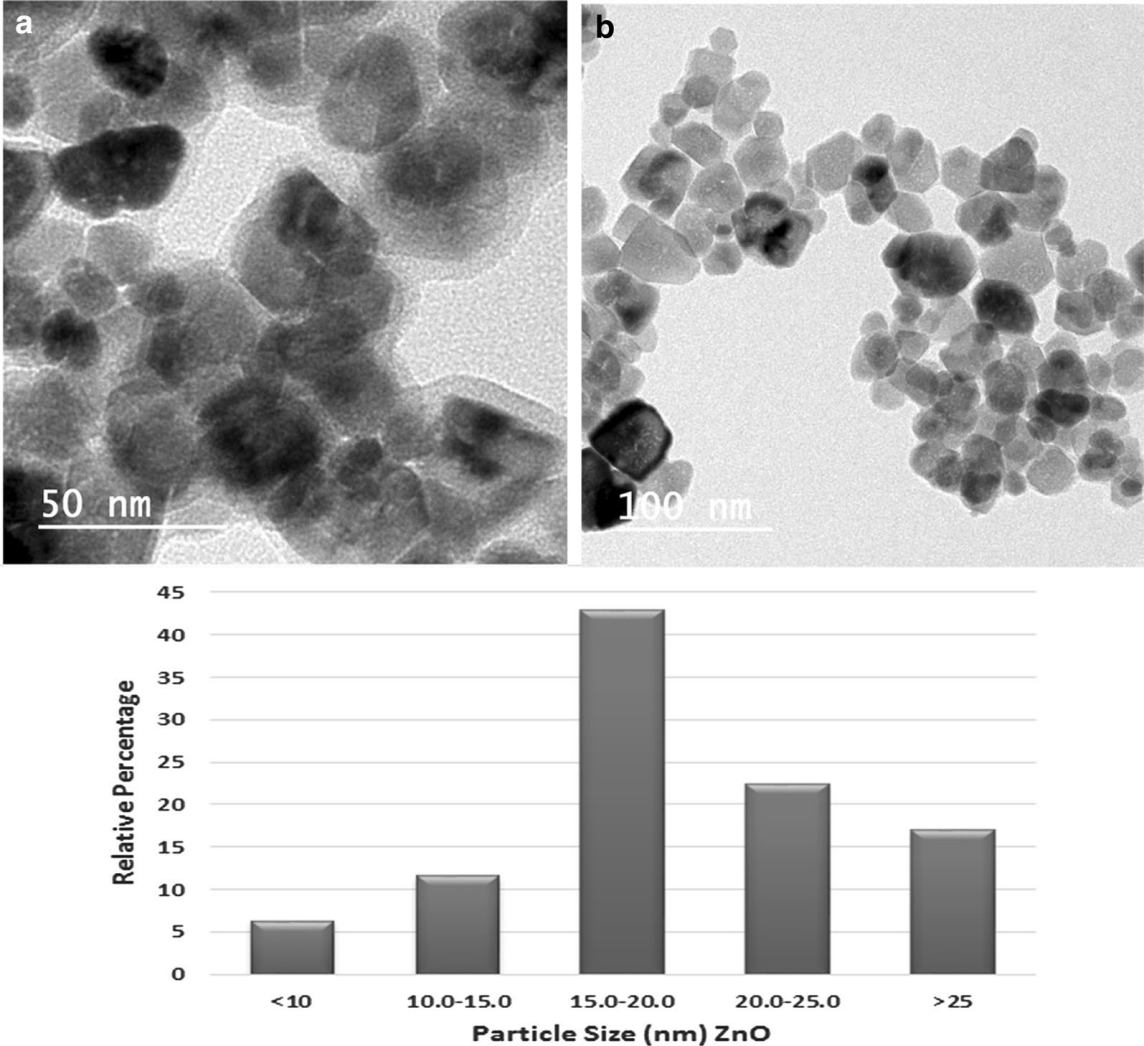
TEM images of ZnO at (**a**) 50 nm (**b**) 100 nm scale with *histogram* showing particle size distribution

**Fig. 4:**
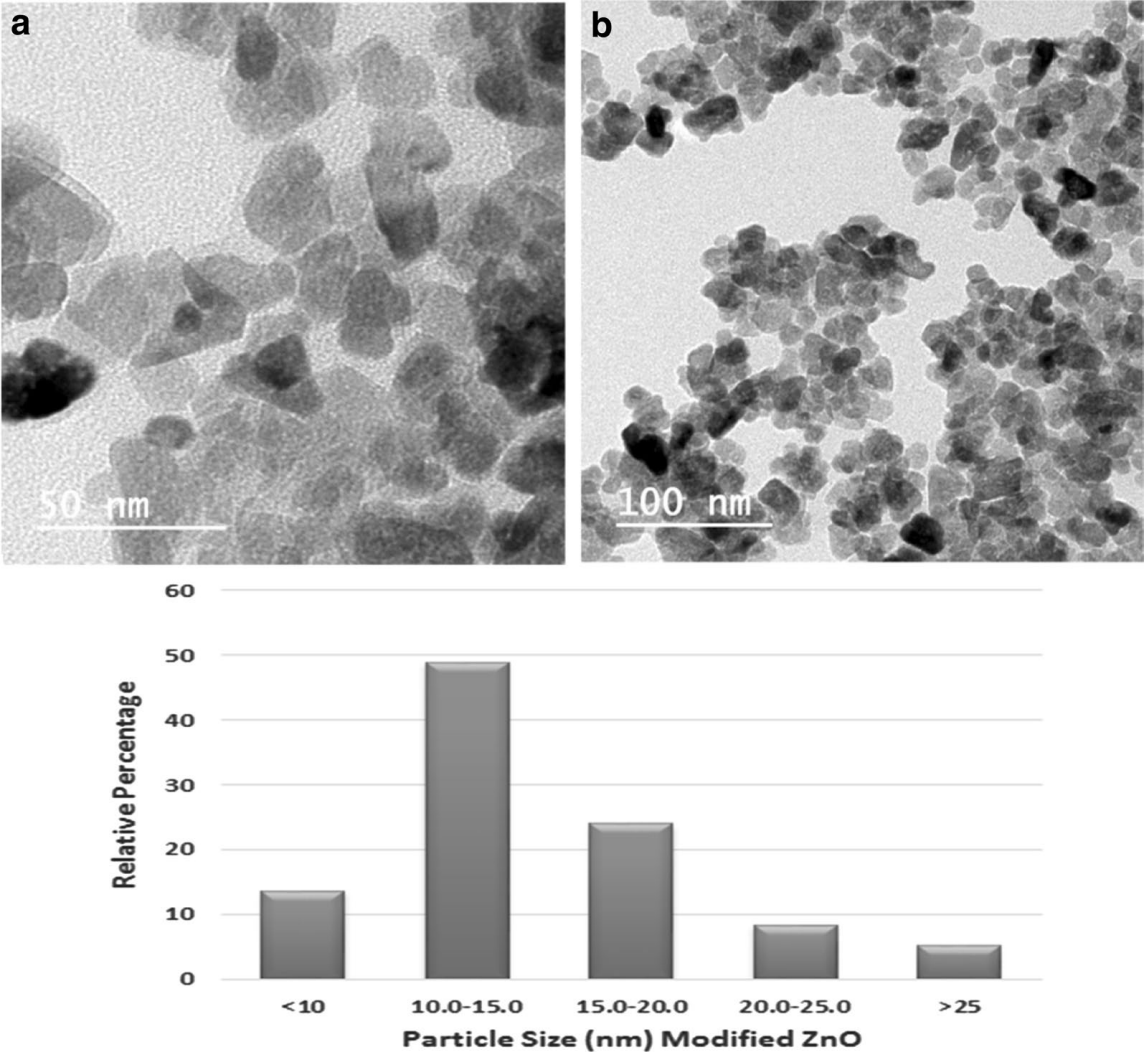
TEM images of modified ZnO at (**a**) 50 nm (**b**) 100 nm scale with *histogram* showing particle size distribution

For morphological characterization of NPs, TEM images as in Figs. 3 and 4 were obtained which shows clear distinction of particle size reduction in case of surfactant used. TEM micrograph of ZnO in Fig. 3 shows clear polygonal structures whereas in case of modified ZnO in Fig. 4, quasi-spherical particles were seen. This is consistent with our result from XRD which shows less crystallinity of modified ZnO than unmodified one. Average particle size distribution of ZnO from TEM histogram was on 15–20 nm and for modified ZnO was on 10–15 nm which was also consistent with XRD results. Polygonal shaped morphology was in accordance with crystalline Wurtzite structure of ZnO [15].

**Fig. 5:**
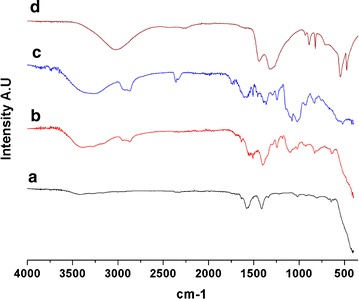
FT-IR Spectra of (*a*) ZnO, (*b*) modified ZnO, (*c*) TritonX only and (*d*) Zinc Acetate Dihydrate from 350 to 4000 cm^−1^. Distinct peak of inorganic divalent metal oxide was seen below 500 cm^−1^ in both ZnO and modified ZnO. Comparative peaks were observed between Triton X-100 only and modified ZnO. Peaks corresponding to 1200–1600 cm^−1^ are of symmetrical and asymmetrical stretching of C=O bond which were found significantly reduced in synthesized particle with respect to Zinc Acetate Precursor. Also, 3500 cm^−1^ peak intensity for bonded −OH are significantly reduced conferring samples are of high purity

FT-IR analysis in Fig. 5 showed a series of absorption peaks. In case of zinc acetate dihydrateprecursor, broad peak was seen around 3000 cm^−1^ which was because of bonded −OH group. Peaks at 1400–1600 cm^−1^ were due to symmetrical and asymmetrical stretching of carboxyl (−COO) group. Peak at 400–500 cm^−1^ suggest divalent metal oxide bond which verified ZnO formation [16]. Comparing the precursor and ZnO powder, a significant reduction in peak intensities at 1400–1600 cm^−1^ was observed. This suggests significant decrease in carboxyl group in the synthesized compound. Hydroxide (−OH) peak at 3000–3500 cm^−1^ range was also completely absent. No impurities peaks were observed in synthesized particles. In modified ZnO, characteristic peak of divalent metal oxide can be observed in accordance with unmodified ZnO with additional peaks similar to TritonX-100 which strongly suggests modification of synthesized NPs.

### Cytotoxicity study

#### Both ZnO and surface modified ZnO shows preferential cytotoxicity

**Fig. 6:**
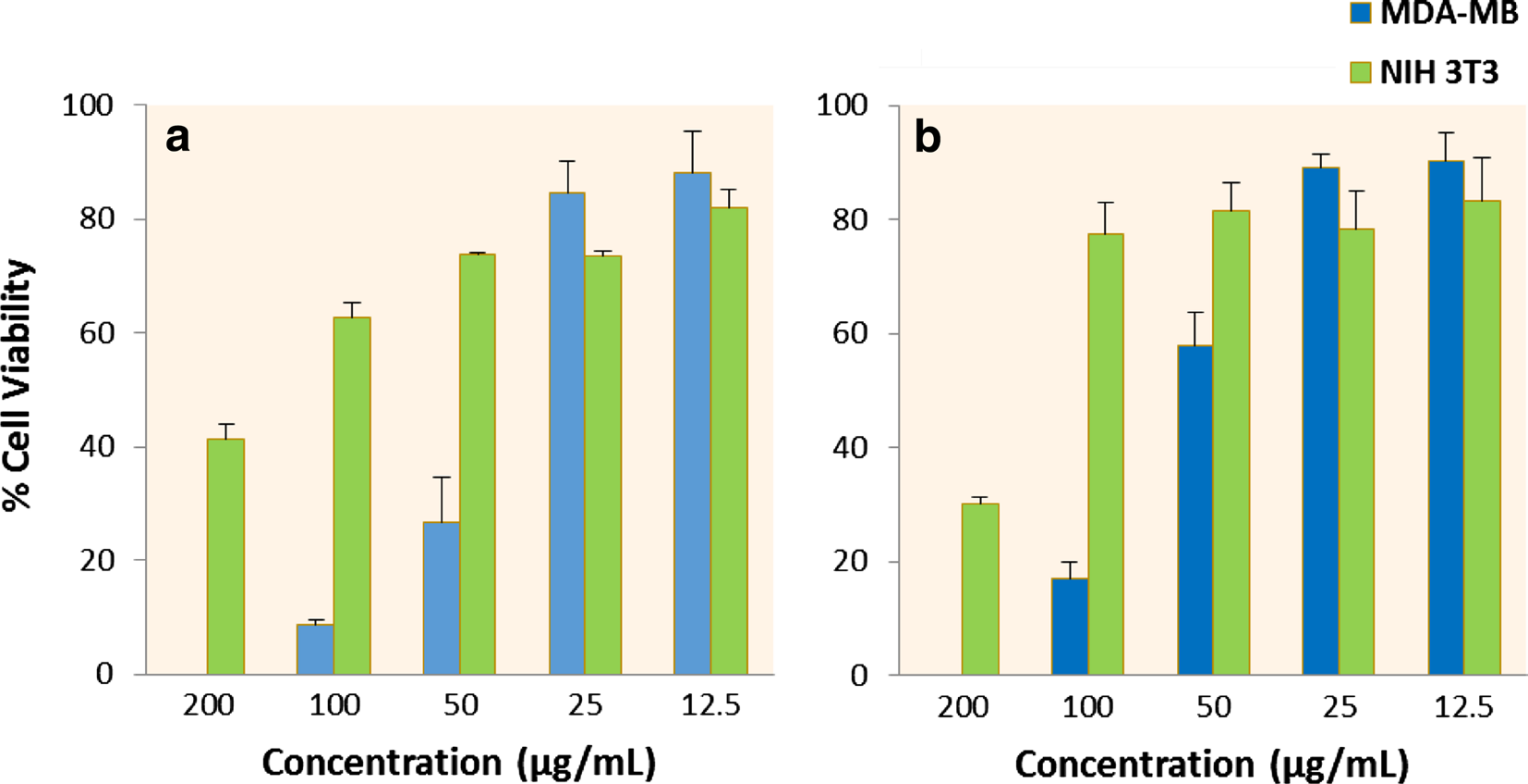
Mean cell viability of (**a**) ZnO and (**b**) modified ZnO treatment on MDA-MB-231 and NIH 3T3. Both cells were treated with ZnO NPs at concentration gradient from 200 to 12.5 μg/mL for 24 h. Corresponding absorbance reading ofdissolved formazan was taken after its conversion by viable cells which was plotted with respect to control (untreated cells) against concentration gradient. Mean ± SE *plot* shows concentration dependent toxicity for MDA-MB-231 cell whereas no strong positive correlation was found in normal NIH 3T3 cell. Percent viability was also found relatively higher in case of normal NIH 3T3 than MDA-MB-231 at given concentration. Two factor ANOVA with α = 0.05 was performed for effectiveness of ZnO NPs between two cell lines and p value for interaction was found to be less than 0.05 verifying difference in effectiveness of NPs on these two cell lines

Result of MTT assay was used to determine percentage cell death with respect to control (untreated cells) as a function of absorbance of dissolved formazan produced from conversion of MTT dye by the action of mitochondrial dehydrogenase enzyme [17]. Figure 6 shows both modified and unmodified ZnO NPs show preferential cytotoxicity against MDA-MB-231 compared to NIH 3T3. Two factor ANOVA with replication was performed at α = 0.05 to analyze variance in effectiveness of concentration gradient of NPs on two cell lines. Results shows *p* value for interaction was less than 0.05 for both ZnO and modified ZnO that reject null hypothesis of equal variance between effects on MDA-MB-231 and NIH 3T3 which justify that effectiveness of concentration gradient of both NPs is different for these two cell lines. This differential cytotoxicity has often been described as selectivity of nanoparticles [18].

#### Cytotoxicity of NPs also depends on surface characteristic, not only on size

**Fig. 7:**
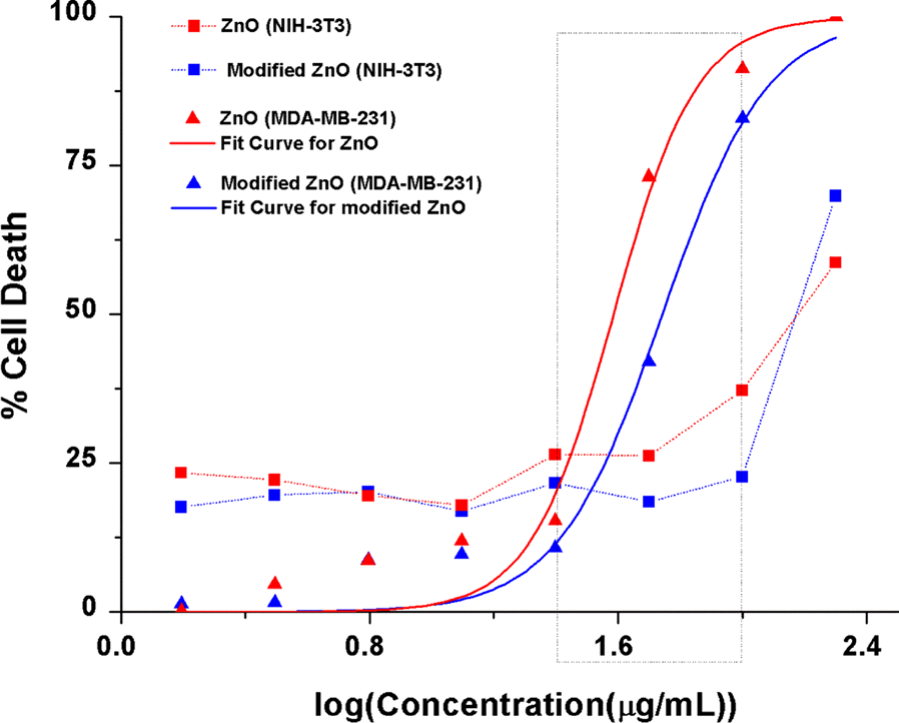
Cytotoxic effects of ZnO and modified ZnO on two cell lines. Non-linear dose response fit for MDA-MB-231 (Adj. R^2^ = 0.979) showing strong correlation of cytotoxicity with increasing concentration. *Plot* for normal NIH 3T3 cell shows concentration independent effect of both particles below 100 µg/ml. Two factor ANOVA was used with α = 0.05 for comparing effects of both NPs on NIH-3T3 which gives p value of`0.001 < 0.05 signifying enhancement of selectivity by biocompatible polymer

Cytotoxic effect of NPs on MDA-MB-231 was found to be concentration dependent as shown in Fig. 7 with Adj. R^2^ of 0.97. The EC50 value of ZnO NPs for MDA-MB-231was found to be 38.44 µg/ml whereas that of modified ZnO NPs was found to be 55.24 µg/ml. While comparing variance of results obtained for ZnO NPs and modified ZnO NPs fitted under the same function using F-test, p value was obtained less than 0.05 which signifies that the effect of TritonX-100 on cytotoxicity of ZnO NPs is statistically significant. TritonX-100 modified ZnO NPs, owing to its smaller size, should have instigated more cytotoxic effect [19] but a contradictory result was observed. One likely explanation for this effect is the coating of reaction site of ZnO NPs by biocompatible TritonX-100 which altered its cytotoxic property. This unexpected result provides strong foundation for the conclusion that the effect of surfactant is pronounced as synergy of its influence on two critical properties: size and surface modification rather than acting singularly on size and influencing cytotoxicity accordingly [20]. The different but comparable cytotoxic effects of ZnO NPs and modified ZnO NPs imputes that surface properties also plays important role in cytotoxicity of NPs along with its size.

No positive correlation was found between cytotoxicity and increasing concentration of stress at given concentration range for NIH 3T3 (p = 0.0019 < 0.05). Although the effect of both NPs on NIH 3T3 is significantly less and pronounced in a concentration independent manner from 12.5 to 100 µg/ml, effect of each particle on NIH 3T3 was found to be significant which was validated by results from two factor ANOVA between ZnO and modified ZnO NPs on NIH 3T3 up to 100 µg/ml that shows p value > 0.05 for within group (concentration gradients) and p value < 0.05 for between groups (ZnO and modified ZnO NPs). Between concentration range 25–100 µg/ml, percentage cell death after treatment with modified ZnO NPs in NIH 3T3 was less than that in treatment with unmodified NPs up to 20 %. This observation is on the agreement with the biocompatibility nature of TritonX [21].

Addressing above findings, it is evident that unmodified particles are more potent than modified particles on MDA-MB-231 but this potency also extends during its treatment in normal cell lines. This means that although the effect on cancer cell line may be greater in case of unmodified particles, effect on normal cell line is also greater. On the other hand, modified particles although prove to be less potent, their effect on normal cell line is even less. Since therapeutic significance of NPs cannot be solely judged by its effect on cancer cell line, but rather should be analyzed through its comparative effect on normal and cancer cells, possible application of TritonX-100 modified ZnO NPs as therapeutic agent holds better promise than unmodified ZnO NPs.

#### Crystal violet staining and DNA fragmentation showing cytotoxicity on NPs treated cancer cells possibly via apoptosis

**Fig. 8:**
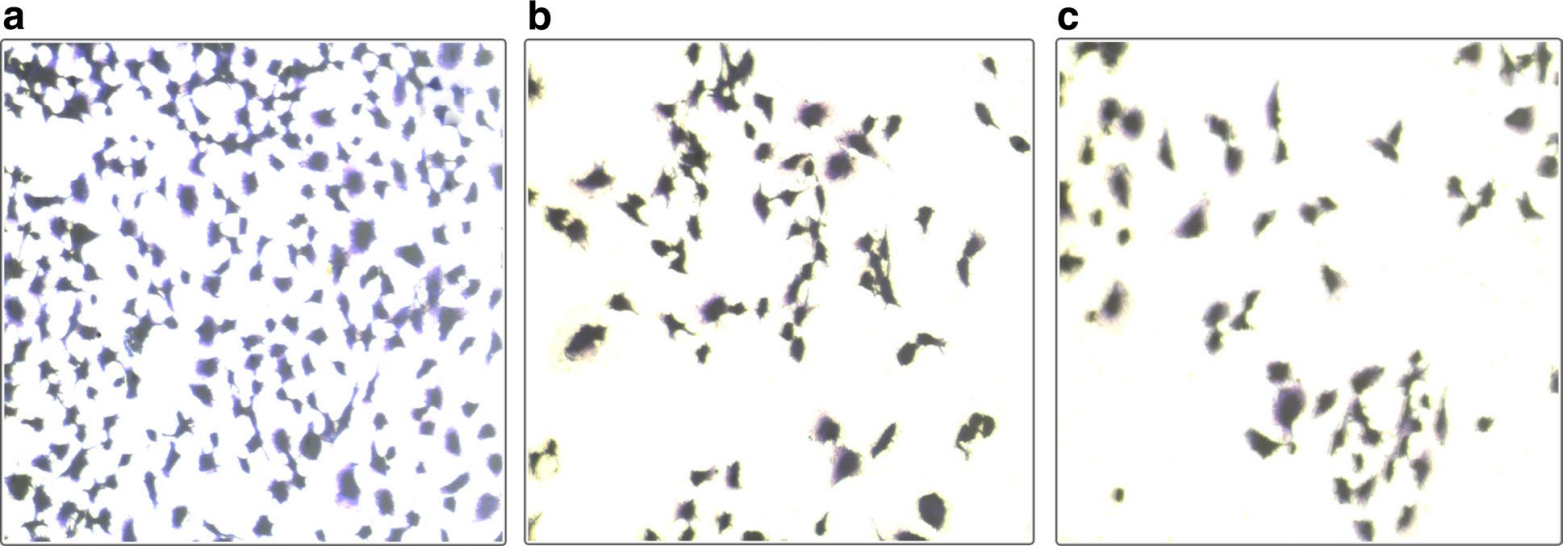
CV staining images of (**a**) Control (untreated) cells, (**b**) ZnO NPs treated cells and (**c**) modified ZnO NPs treated cells (MDA-MB-231) after 24 h treatment of stress. Cells were fixed by 4 % paraformaldehyde for 30 min, stained by 0.05 % Crystal violet for 30 min and then subsequently washed with distilled water followed by microscopic observation at 10X. This observation clearly shows less number of viable cells in treated cells than untreated control

Apoptotic cells show distinct morphological and biochemical hallmarks. Some of them include cell shrinkage, chromatin cleavage, nuclear condensation and disintegration, formation of pyknotic bodies, etc. [22]. Crystal violet dye (Hexamethylpararosaniline) is a mixture of violet rosanilins that stains nucleus dark blue and cytoplasm light blue. In solution, it dissociates into ions which upon entering the cell binds preferentially with negatively charged components, typically DNA where two adjacent A-T residues occur [23]. Since CV stains viable cells, reduction of viable cells in treated cells as seen in Fig. 8 in comparison to untreated cells verified the MTT result of cell cytotoxicity by NPs.

**Fig. 9:**
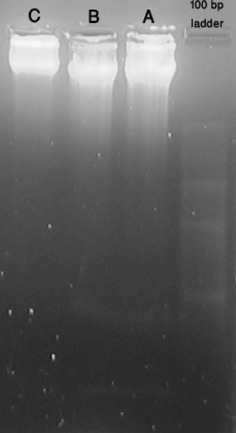
UV-Illumination of fragmented DNA bands of ZnO NPs and modified ZnO NPs treated cells in 1.2 % Agarose gel along with control untreated cells (*leftmost*) and 100 bps ladder (*rightmost*) segregated on 5 V/cm for 2 h

DNA fragmentation pattern is distinct in apoptotic cells creating laddering effect in contrast to necrotic cells in which random DNA fragmentation occur creating smear rather than ladder in gel [24]. Figure 9 shows UV-illuminated gel of whole DNA extracted from treated and untreated cells with DNA ladder of 100 bp at rightmost position. Distinct bands of fragmented DNA can be observed in case of treated cells (both with ZnO and modified ZnO NPs). But no such band observed for untreated cells. This suggests that both modified and unmodified ZnO NPs induce DNA fragmentation within cells, supporting induction of apoptosis intracellularly [8, 25] and confirms that surface modification had not changed this basic mechanism of cell death.

## Experimental

### Particle synthesis

Zinc acetate dihydrate, sodium hydroxide and methanol were purchased from Sigma Aldrich. ZnO NPs were synthesized using precipitation technique as described in [26]. 0.25 MNaOH was added to 0.05M zinc acetate containing 0.08 M TritonX-100 surfactant under vigorous stirring at room temperature, the cloudy viscous sediment thus obtained was filtered using 42-grade filter paper under vacuum filtration, dried overnight at 50 °Cand then calcinated at 200 °C for 2 h in muffle furnace. In another setup, all above procedure was followed except no TritonX-100 was used.

### Characterization

XRD and TEM were carried out at IIT, Roorkee, India. X-ray diffraction spectra were recorded at 0.154 nm wavelength (λ) of Cu-kα radiation using Rigaku-Geiger diffractometer with range of 2θ from 5° to 80°. Phase identification and crystallographic planes were determined by comparing peak positions with reference JCPDS file. Particle size was computed using Scherrer’s equation: D = Kλ/βcosθ, where K = 0.9 is shape factor, β is FWHM in radians and θ is Bragg’s angle [27]. For TEM analysis, required volume of sample was sonicated in acetone (1 %, w/v) and applied over carbon coated copper grid. TEM images were recorded at 10-KX magnification and 0.2-μm scale over JEOL 1011 (Tokyo, Japan) at 80 kV. Similarly, FT-IR spectroscopy was performed on powder samples and precursors using Shimadzu IR Prestige 21 FT-IR Spectrometer and corresponding spectrum was generated using IR-Solution software.

### Cell culture

Two cell lines; normal mouse fibroblast NIH 3T3 and human breast adenocarcinoma MDA-MB-231 were maintained at Kathmandu University while Dulbecco’s modified Eagle medium (DMEM; Life Technologies, USA), fetal bovine serum (Sigma, Germany), penicillin/streptomycin (Sigma, Germany), MTT dye (Amresco, USA), Trypsin (Amresco, USA), and Amphotericin “B” (Sigma, Germany) were purchased. Cell lines were maintained in DMEM cell culture medium supplemented with 10 % FBS, 0.5 % antibiotic solution (penicillin and streptomycin stabilized with glutamine), 0.5 % antimycotic solution (amphotericin “B”) at 37 °C supplemented with 5 % CO_2_ [28]. Stock solutions of both ZnO NPs (with and without TritonX-100) were prepared in Dulbecco’s phosphate buffer saline (pH 7.4 ± 0.1). These solutions were then sonicated in water bath sonicator (40 min) prior to use to prevent particle aggregation.

### Cell viability assay

MTT assay as mentioned by [28] was performed. Log phase cells were harvested and seeded in 96 well plates at density of 10,000 cells per well. After 24 h of attachment, the media was replaced with fresh media supplemented with NPs at concentration range from 200 to 1.5625 µg/ml via serial dilution. After 24 h of treatment, cells were washed with DPBS twice and 10 µl of 5 mg/mL MTT dye was added. It was incubated for 4 h and reading was taken at 570 nm with background subtraction of 630 nm band pass filter. Percent cell viability was expressed as percent relative absorbance of sample with respect to control and percentage death as percent relative difference.

### CV staining

Cultured cells were seeded in 12 wells plate at density of 80,000 cells per well and incubated. After 24 h, cells were treated with NPs at 40 µg/ml for ZnO and 55 µg/ml for modified ZnO for another 24 h. Non-adherent cells were washed off using DPBS and remaining cells were fixed with 4 % paraformaldehyde for 30 min. Then 0.05 % of crystal violet solution in 20 % ethanol was added and left to stain for 30 min. Finally, excess stain was washed off using distilled water and observed on phase contrast microscope at 10X magnifications.

### DNA fragmentation assay

Apoptotic DNA ladder kit was purchased from Invitrogen (Ref. KHO1021). MDA-MB-231 cells maintained in T_25_ flask were trypsinized and seeded in 6 wells plate at density of 80,000 cells per well. After 24 h, ZnO NPs were added at same concentrations mentioned in CV-staining. After another 24 h of treatment, media containing dead non-adhered cells were directly collected in falcon tube whereas adhered cells were trypsinized and collected. Thus obtained solutions were centrifuged at 1000 rpm for 5 min and clear cell pellets were obtained with repeated washing and centrifuging with DPBS. DNA was then isolated using purchased kit protocol. 30 μl of each extracted DNA sample was loaded onto a 1.2 % Agarose gel containing 0.5 μg/ml EtBr. The gel was run at 30 Volts for 2 h and EtBr-stained DNA was visualized by trans-illumination with UV light and photographed.

### Statistical analysis

All statistical analyses were done at level of significance 0.05. Dose-response curve was fitted under inbuilt function for dose-response in Origin8 Pro software using non-linear curve fitting model. Two-factor ANOVA for studying variance between and within samples was applied using Microsoft Excel 2010.

## Conclusions

Easy and controlled synthesis of ZnO NPs posing preferential cytotoxic property can be achievedat less than 20 nm size using simple precipitation techniques with no impurities. Comparable morphology and crystallinity of nanoparticles was achieved by in situ use of surfactant. Our results showed that use of surfactant decreased particle size possibly by coating surface and providing steric hindrances during particle growth but at the same time made surface more biocompatible thereby providing antagonistic effect between size and surface property on toxicity. This can be observed in dose response curve of ZnO and modified ZnO on MDA-MB-231 where modified ZnO has more EC50 values of 55.24 µg/ml than unmodified, 38.44 µg/ml making it less potent. While there was concentration dependent cytotoxicity of NPs on cancer cell, no positive correlation was found between normal cell cytotoxicity and increasing concentration of stress up to 100 µg/ml. Our research directs that ZnO NPs shows effective preferential cytotoxicity at a concentration range of 25 to 100 μg/mL with enhanced preferential cytotoxicity shown by the modification of TritonX-100. Distinct fragmented band observed in DNA fragmentation assay signifies that the mechanism of cytotoxicity on cancer cell is apoptosis. This result of enhanced preferential cytotoxicity by biocompatible polymer modified NPs can be exploited to make efficient anticancer agent comparing toxicity on both cancerous cell and normal proliferating cells.
